# A case of urinary bladder agenesis and bilateral ectopic ureters: a case report

**DOI:** 10.1186/s12894-018-0396-6

**Published:** 2018-09-26

**Authors:** Iman Ibrahim Nazer, Ghufran Alhashmi, Sara Nawfal Sharief, Nada Abdullatif Hefni, Abdulrahman Ibrahim, Sherif M El-Desoky, Ahmed Jalal Alsayyad, Osama Yousef Safdar, Jameela A Kari

**Affiliations:** 1 Pediatric department and Pediatric Nephrology Center of Excellence, King Abdulaziz University, PO Box 80215, Jeddah, 21589 Kingdom of Saudi Arabia; 2 Department of Pediatrics, Jeddah, Kingdom of Saudi Arabia; 3 Department of Urology, Jeddah, Kingdom of Saudi Arabia; 4 Department of Radiology, Jeddah, Kingdom of Saudi Arabia; 5 King Abdulaziz University (KSA), Jeddah, Kingdom of Saudi Arabia

**Keywords:** Bladder agenesis, Ectopic ureters, Ambiguous genitalia, Urogenital, Urogenital anomalies

## Abstract

**Background:**

Urinary bladder agenesis is a very rare congenital anomaly with very few cases reported in the literature.

**Case presentation:**

We report a one-month-old baby presenting with ambiguous genitalia and recurrent urinary tract infections. Her clinical course was complicated by renal impairment. Magnetic resonant imaging (MRI) revealed a diagnosis of bladder agenesis with bilateral ectopic insertion of the ureters into the vagina, associated with several other anomalies. The patient underwent bilateral high anterior ureterostomies in an hospital abroad at 5.5 months of age. She then developed ureteral necrosis that had to be corrected with left pyeloplasty and by placing a left nephrostomy tube for drainage. Eventually, the patient’s renal function declined, and she developed chronic kidney disease (CKD).The case with its imaging findings and pathogenesis as well as a review of the literature are presented.

**Conclusions:**

Urinary bladder agenesis is a rare congenital condition that can be associated with multiple anomalies. Early diagnosis and therapeutic intervention can prevent progression to chronic kidney disease.

## Background

Anomalies of the urogenital system are commonly found on pre- and postnatal imaging studies. Urinary bladder agenesis is one of the rarest urinary tract anomalies, with a reported incidence of 1 in 600,000patients [1]. Only 64 cases have been reported worldwide, with a significant female predominance, where it occurs thirty times more often in girls than in boys [2–5]. Only 25 live births have been reported, as the condition is usually associated with other severe malformations that are incompatible with life^6^. Reported associated anomalies include those of the urogenital, gastrointestinal, vascular and musculoskeletal systems [2, 6]. The cause of urinary bladder agenesis has been attributed to urogenital sinus injury at weeks 5–7 of embryogenesis [4]. We report a one-month-old girl with urinary bladder agenesis and bilateral ectopic insertion of the ureters into the vagina in association with urogenital, anal, vascular and skeletal anomalies.

## Case presentation

The patient was the product of a term uncomplicated pregnancy and a spontaneous vaginal delivery, who was found to have ambiguous genitalia after birth in another center. The parents are nonconsanguineous and have 5 other healthy siblings. The patient was referred to the plastic surgery unit at our hospital at the age of 1 month for possible surgical correction. She also had low imperforate anus with stenosis that was dilated.

**Fig. 1:**
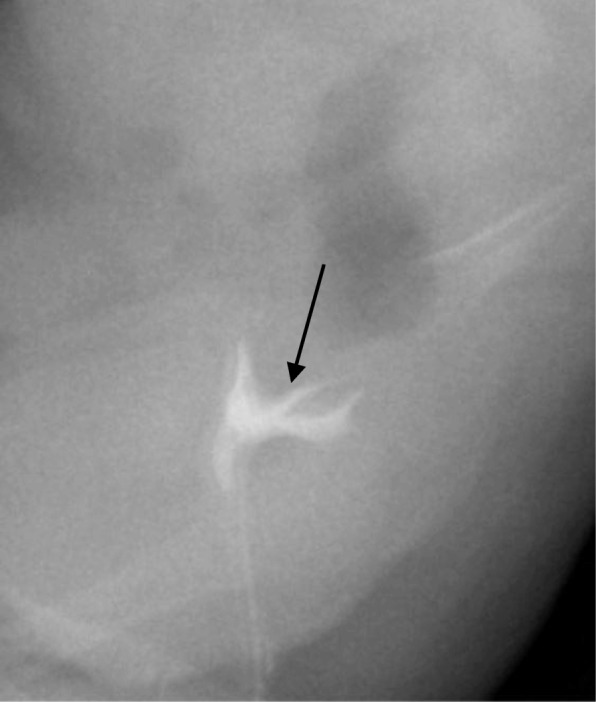
Small amount of contrast was injected through a catheter inserted through the perineal opening anterior to the anus, draining urine. The contrast opacified a triangular shaped cavity, with a uterine cervix impression at its superior aspect (arrow). The urethra was not opacified

Laboratory investigations ruled out underlying congenital adrenal hyperplasia and revealed a female karyotype of 46 XX. Cystoscopy revealed what appeared to be a short urethra and atresia of the lower vagina. An ascending urethrogram then followed, with the aim of assessing the location of the confluence between the vagina and urethra. The urethrogram failed to opacify the urethral canal; instead, it revealed a triangular-shaped cavity with a uterine cervix impression at its superior aspect (Fig. 1). The plan was to further evaluate with MRI as an outpatient to better detail the urogenital anatomy.

**Fig. 2:**
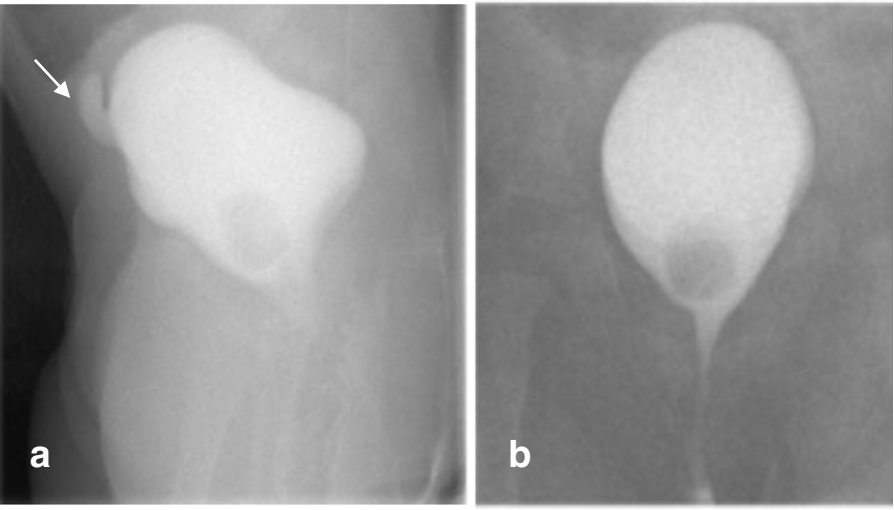
**a** Lateral and (**b**) AP projections of MCUG: Contrast opacified a pear-shaped structure in the pelvis at the location of the urinary bladder, with a tubular outpouching from its superior aspect (arrow). The contrast filled structure was thought to represent a urinary bladder with a diverticulum or reflux into an ectopic ureter. After assessment with MRI this structure was the vagina, and the outpouching was an ectopic ureter draining to the anterior wall of the vagina

The patient then suffered from recurrent urinary tract infections (UTIs) indicated by several positive urine cultures, the first of which was associated with dehydration and acute kidney injury (AKI), requiring admission. On her initial ultrasound, the left kidney was normal, the right kidney was dysplastic and it was difficult to assess the urinary bladder. A micturating cystogram (MCUG) showed a urinary bladder-like structure, with a tubular outpouching from its superior aspect, thought to be a diverticulum or a refluxing ectopic ureter (Fig. 2).

**Fig. 3:**
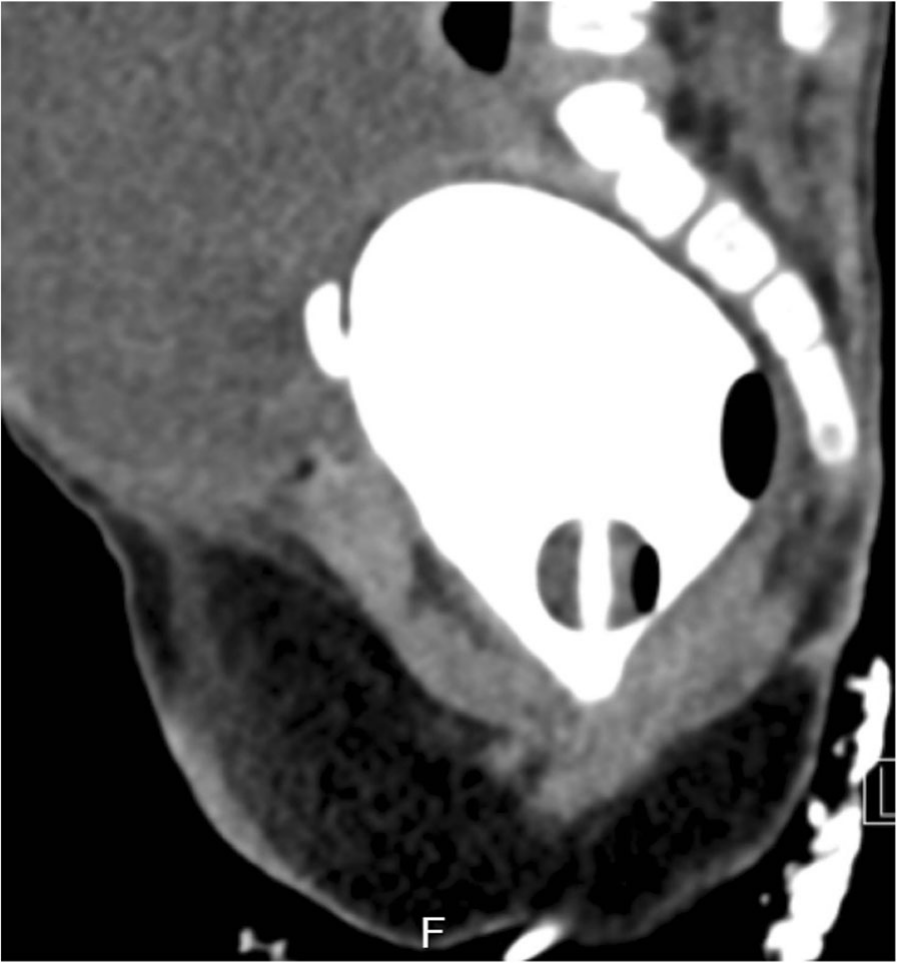
CT cystogram, showing no leak of contrast. The contrast opacified structure had similar appearance to that seen on the MCUG, however, its location was thought to be lower and more posterior than the normal location of a full urinary bladder. Thus this structure was thought to possibly represent the vagina instead

**Fig. 4:**
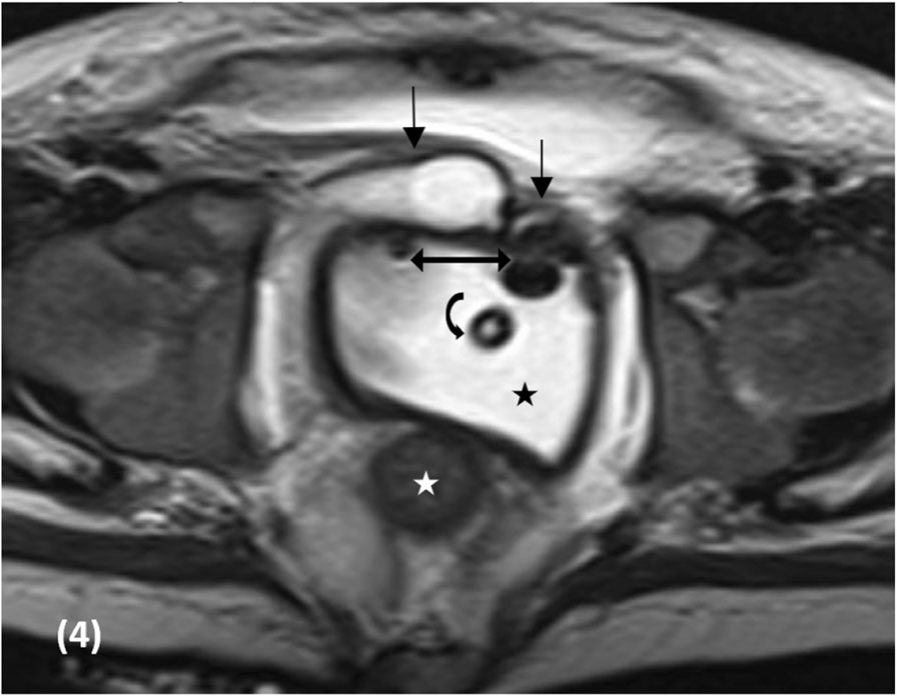
Axial T2 MRI of the pelvis at the level of the rectum (star), showing abnormal anteriorly directed bilateral distal ureters (black arrows), lying superficially posterior to the anterior abdominal wall, inserting into the vagina, close to the midline. The vagina is distended with fluid (black star) and contains a Foley catheter (curved black arrow). There are two foci of air in the vagina (double sided arrow)

**Fig. 5:**
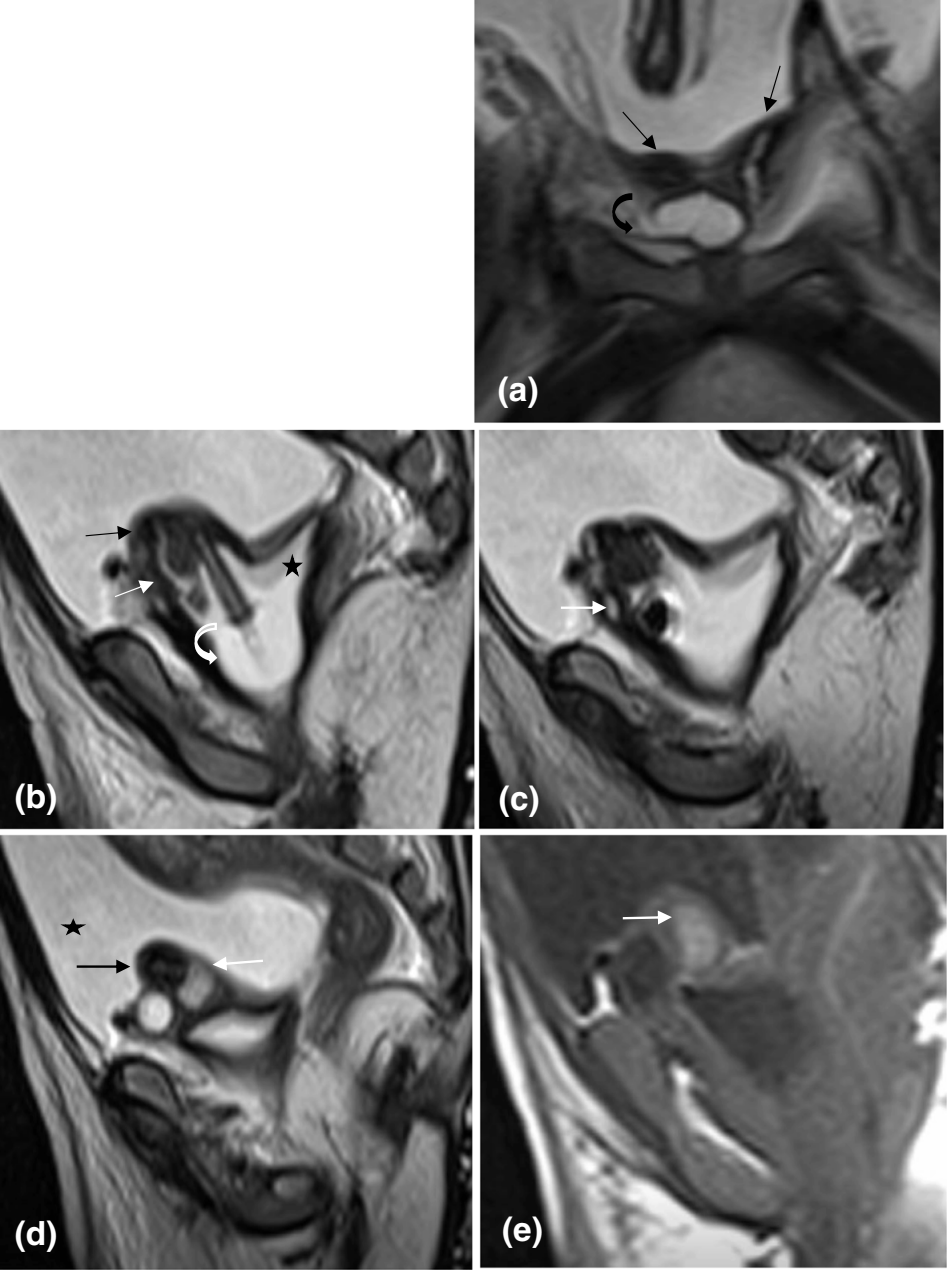
**a** Coronal T2 MRI of the pelvis showing two uterine bodies (black arrows) and cervices (not shown), in keeping with uterus didelphys. The dilated distal right ureter (curved arrow) lies anterior to the uterus (**b**) Left parasagittal T2 MRI of the pelvis showing the left uterine body (black arrow) and cervix (white arrow) opening into the vagina. Fluid (star) and a Foley catheter with a balloon (curved arrow) are seen in the vagina

**Fig. 6:**
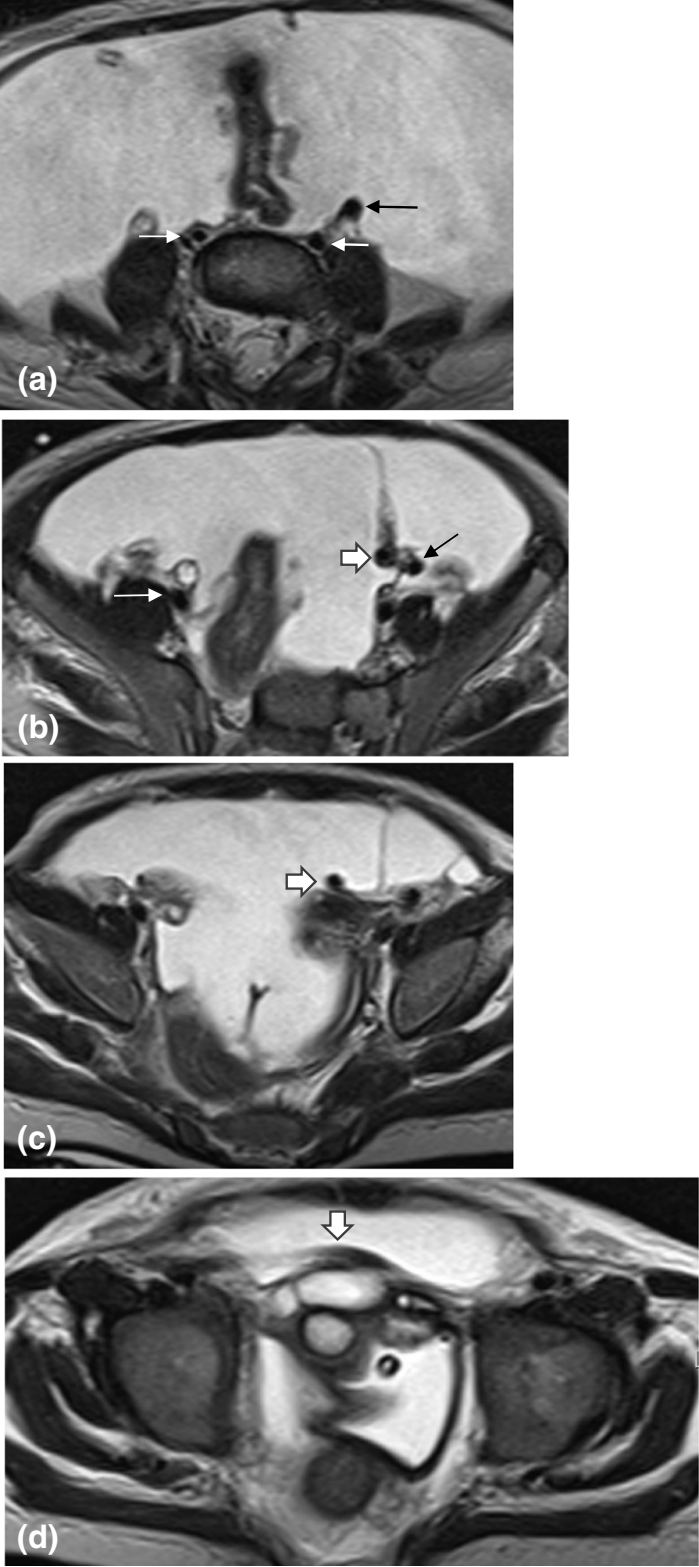
**a** Axial T2 MRI of the abdomen at the level of the common iliac vessels, showing bilateral common iliac veins (white arrows). The left common iliac artery is present (black arrow). However, the right is absent. **b**, **c** and **d** More inferior axial T2 images at the level of the external iliac vessels showing the right external iliac vein (white thin arrow), without an accompanying artery. The left external iliac artery (black arrow), gives rise to a large vessel (thick white arrow) that crosses to the right side and passes superficially just posterior to the anterior abdominal wall, anterior the insertion of the distal ureters. This artery then continued as the right common femoral artery. The internal iliac vessels were absent bilaterally

At 5 months of age, the baby developed progressive ascites. Ascitic taping and drainage showed high ascitic fluid creatinine level compared to serum creatinine level (220 μmol/L compared to 85 μmol/L, respectively), denoting urinary ascites. CT cystogram performed to rule out bladder injury showed no leak of contrast. The structure opacified with contrast on the CT cystogram had a similar appearance to that observed on the MCUG. Nevertheless, the structure was lower and more posterior than was the normal full urinary bladder; hence,it was thought to possibly be the vagina (Fig. 3). Evaluation by magnetic resonance imaging (MRI) followed to assess for urine leak with the use of delayed post contrast MR urography and to detail the anatomy of the urogenital system. MR confirmed that the structure seen on both CT cystogram and MCUG studies was in fact the vagina with ectopic insertion of the ureters into the anterosuperior aspect of the vagina. It also showed urinary bladder agenesis, uterus didelphys with unilateral hematocolpos (Figs 4 and 5). In addition, there was an unusual course of the abdominal aorta, with a low bifurcation in the left iliac fossa, giving rise to two large arteries. The left branch continued as the left external iliac artery, while the right branch crossed the midline to supply the right lower limb. While crossing the midline, the right branch had a superficial course behind the lower anterior abdominal wall, anterior to the insertion of the ureters into the vagina (Fig. 6). There were no internal iliac vessels bilaterally. Segmentation anomalies of the lumbar spine were also seen on the MRI.

Surgery was then planned to perform bilateral nephrostomy tubes for urine diversion and to obtaina better idea about anatomy. Before the surgery, the urologist was aware of the urogenital anomalies and the superficial course of the artery supplying the right lower limb seen on the MRI. However, the parents decided to take the baby to another center abroad for treatment and operation, where she had bilateral high anterior ureterostomies at 5.5 months of age.

She presented 3 weeks later to our emergency department with AKI, ascites and left ureterostomy obstruction. At presentation, the ureterostomies were nonfunctional, and insertion of the nephrostomy tube was deemed impossible according to the interventional radiologist. Therefore, surgical exploration of the left side (the only functioning side) was performed, revealing complete ureteral necrosis and a 1-cm segment of renal pelvis remaining. This renal pelvis remnant was open to the peritoneal cavity, and there was intraperitoneal urine leakage. The left renal pelvis was then closed after leaving a nephrostomy tube in place for drainage. No intraoperative or postoperative bleeding was encountered. The nephrostomy tube was then complicated by frequent blockage, leakage and infection with difficult reinsertions. The patient received several types of antibiotics throughout her hospital stay for her recurrent UTIs, including trimethoprim/sulfamethoxazole, imipenem, piperacillin/tazobactam, fluconazole, caspofungin, ceftazidime, colistin, nitrofurantoin, ciprofloxacinand clindamycin.

The patient’s renal function declined slowly, and at the age of 11 months, she had evidence of chronic kidney disease associated with high creatinine, hyperphosphatemia, metabolic acidosis and anemia.

## Discussion

We present a case of bladder agenesis associated with several other congenital anomalies. To our knowledge, this is the 26^th^live-birth case of bladder agenesis reported in the literature.

The underlying etiology remains indeterminate and was postulated to be due to an insult at the 5th to 7^th^ week of embryogenesis, leading to urogenital sinus injury [3, 7]. Alternatively, the etiologymay be related to lack of urinary bladder distention with urine due to failure of integration of the ureters and mesonephric ducts into the trigone [3, 7].

Age, presentation and outcome are variable and depend on the associated anomalies. Most cases present during infancy or childhood. Only one reported case presented as an adult, at the age of 22 years [8]. There is female predominance, where girls are affected thirty times more than boys; reported cases have all been females except for four males [9–12]. This predominance is explained by the anatomy of the reproductive system of females that enables them to maintain urine drainage, as opposed to male patients who are usually stillborn or die early [2]. Male neonates with absent bladder can survive only if urine drainage is allowed through ectopic ureteric insertion into the rectum or a patent urachus [3, 11, 13]. Similar to our case, patients with bladder agenesis frequently present with recurrent UTIs [6, 7, 13, 14], or with symptoms related to other associated anomalies. Prognosis of these patients is variable but is poor overall. Only one-third of reported cases survive after birth [3, 7, 13, 15].

Our case, as with most reported cases, is a female with a presentation of ambiguous genitalia and recurrent UTIs associated with several anomalies. Specifically, our case had urinary bladder agenesis, bilateral hydroureteronephrosis, ectopic insertion of the ureters into the vagina, dysplastic right kidney, uterus didelphys, low aortic bifurcation with superficial course of the right iliac artery posterior to the anterior abdominal wall, absent internal iliac vessels and a segmentation anomaly of the spine. There was a considerable delay in the patient’s diagnosis with bladder agenesis, as she was diagnosed at the age of 6 months. This delay was due to the rarity of the condition, requiring an advanced center for management. Despite this limitation, we had good radiological tests, a good pediatric nephrology team and an expert pediatric urologist.

Reported cases of urinary bladder agenesis had various associated abnormalities. The most common associated anomalies were those of the urinary system, including renal dysplasia/hypoplasia, pelvic kidneys, crossed fused renal ectopia, horseshoe kidneys, ectopic insertion of the ureters and urethral agenesis [2, 3, 5–7, 10, 13–15]. In females, most ectopic ureters drained to the vagina [2, 8, 14, 16, 17], urogenital sinus [5, 6, 18] orvestibule [7, 13, 19, 20] and rarely to the skin [21]. In males, ureters drained to the anterior wall of the rectum [11, 12], to the seminal vesicles [10] or to the prostatic urethra [9]. Various genital anomalies were also found in association with urinary bladder agenesis, including absent uterus [19], unicornuateand bicornuate uterus [2, 18], vaginal agenesis [22] and penile agenesis [3].

Variations of the aorta and iliac arteries were described in cases with urogenital anomalies. These alterations can predispose major vessels to injury during surgery, leading to major complications such as hemorrhage or limb loss [23]. Therefore, preoperative assessment of the vascular anatomy in these patients can be helpful to avoid such complications. We also observed absence of the internal iliac arteries and veins in our patient, which we did not find in previous reports.

Other described associations include imperforate anus, omphalocele and anomalies of the spine and musculoskeletal systems, including VACTERL association, spina bifida, vertebral segmentation anomalies, scoliosis and hip dysplasia [2, 5, 10, 16, 21, 22].

The diagnosis of bladder agenesis was made by MRI in most reported cases [6, 8–10, 12, 14, 16]. Few cases were diagnosed on CT [2], retrograde ureterogram [7], intravenous urography (IVU) [13, 19] or surgery [11, 18]. These results illustrate the role of MRI in the imaging workup of patients with ambiguous genitalia, as it can detail the anatomy of the urogenital system and assess for possible associated anomalies at the same time. When feasible, it may be better to start imaging these patients with an ultrasound followed by MRI before even attempting MCUG to avoid misleading appearances on MCUG; in our case, the distended vagina simulated the appearance of the urinary bladder on MCUG. Our patient did not undergo dimercaptosuccinic acid(DMSA) scanning because of the recurrent UTIs leaving no time interval between the attacks. Nevertheless, DMSA scanning is very sensitive for diagnosing dysplastic kidneys [24].

## Conclusion

Urinary bladder agenesis is a rare congenital condition that can be associated with multiple anomalies. It should be considered in patients with unexplained or atypical UTIs because early diagnosis and therapeutic intervention can prevent or delay the progression to chronic kidney disease. Early MRI is advised for definitive diagnosis.

## Abbreviations

AKI: Acute kidney injury
DMSA: Dimercaptosuccinic acid
IVU: Intravenous Urography
MCUG: Micturating cystourethrogram
MRI: Magnetic resonant imaging
UTIs: Urinary tract infections
